# Effects of Cold Exposure on Energy Metabolism and Oxidative Stress in Two Plateau Rodents

**DOI:** 10.1093/iob/obag051

**Published:** 2026-08-29

**Authors:** D Hou, Y Wang, W Zhu

**Affiliations:** School of Life Sciences, Yunnan Normal University, Kunming 650500, China; College of Life Science and Technology, Inner Mongolia Normal University, Hohhot 010022, China; College of Life Sciences, Hebei University, Baoding 071000, China; School of Life Sciences, Yunnan Normal University, Kunming 650500, China

## Abstract

Low temperature is a major environmental constraint for animals inhabiting cold plateau regions because it increases thermoregulatory energy demand and can disrupt oxidative balance. We examined physiological responses of two sympatric plateau rodents, *Eothenomys miletus* and *Apodemus chevrieri*, during 28 days of cold exposure (5°C ± 1°C) relative to control conditions (25°C ± 1°C). Both species increased resting metabolic rate (RMR) and food intake and showed lower body mass under cold conditions. Cold exposure also increased liver mass and induced digestive-tract remodeling, with more extensive changes in *E. miletus* than *A. chevrieri*. Activities of the antioxidant enzyme superoxide dismutase (SOD) and catalase (CAT), together with the oxidative stress markers malondialdehyde (MDA) and hydrogen peroxide (H_2_O_2_), changed in a tissue- and species-specific manner. Correlation and principal component analyses further indicated different patterns of oxidative regulation in the two species. *Eothenomys miletus* showed a comparatively dense inter-organ correlation structure centered on CAT, whereas *A. chevrieri* showed fewer significant inter-organ associations. Thus, the two rodents share a broad metabolic response to sustained cold exposure but differ in the organ-level organization of oxidative balance.

## 摘要

对于寒冷高原地区的动物来说, 低温是主要的环境限制因素, 它不仅增加动物体温调节能量的需求, 且可破坏氧化平衡。本文对同域高原啮齿动物大绒鼠 (*Eothenomys miletus)* 和高山姬鼠 (*Apodemus chevrieri)* 在冷暴露28 d (5°C ± 1°C) 与对照 (25°C ± 1°C) 的生理反应进行了研究。在寒冷条件下, 两个物种的静息代谢率 (RMR) 和食物摄入量都有所增加, 而体重显著降低。冷暴露导致肝脏质量增加, 并诱导消化道重塑, 其中大绒鼠的变化比高山姬鼠更为显著。抗氧化酶超氧化物歧化酶 (SOD) 和过氧化氢酶 (CAT) 的活性以及氧化应激标记物丙二醛 (MDA) 和过氧化氢 (H_2_O_2)_ 的变化具有组织和物种特异性。相关分析和主成分分析进一步表明, 这两个物种的氧化调节模式不同。大绒鼠表现出以CAT为中心的较密集的器官间关联结构, 而高山姬鼠表现出较少的器官间关联。因此, 这两种啮齿动物对持续冷暴露具有显著的代谢反应, 但在器官水平的氧化平衡组织上存在差异。

## Introduction

Low temperature is an important environmental factor limiting the distribution and population dynamics of animals in cold plateau regions, and it imposes a high thermoregulatory cost on small endothermic mammals. Small rodents commonly increase resting metabolic rate (RMR) and food intake to defend body temperature, sometimes at the expense of body mass (Wang and Wang 1996; Chi and Wang 2011; Gordon 2012). Chronic cold exposure can also induce hypertrophy of metabolically active organs and remodeling of the digestive tract, thereby increasing the capacity for nutrient processing and sustained heat production (Chen et al. 2014). This resulting increase in metabolic flux can increase reactive oxygen species (ROS) production and disturb the balance between ROS generation and clearance (Dickinson and Chang 2011). If this imbalance persists, oxidative damage can accumulate despite compensatory antioxidant responses (Selman et al. 2013; Marri and Richner 2015). Antioxidant enzymes, particularly superoxide dismutase (SOD) and catalase (CAT), are central components of this defense because they limit superoxide- and hydrogen-peroxide-associated oxidative injury (Jomova et al. 2023; Jomova et al. 2024). Accordingly, changes in malondialdehyde (MDA) and hydrogen peroxide (H_2_O_2_) can provide complementary information about the residual oxidative cost of cold acclimation (Jomova et al. 2023).

Temperature-induced changes in energy expenditure do not produce uniform oxidative responses across species or tissues. In rats, cold exposure has been associated with increased lipid peroxidation in the brain and increased SOD activity in the heart and kidneys, whereas other tissues show unchanged or reduced oxidative indices (Lomakina 1980; Vasilijevic et al. 2007; Yuksel et al. 2008). Heat exposure can likewise reduce antioxidant capacity in specific tissues, including the testes (Kanter et al. 2013). Among small rodents, cold acclimation increases antioxidant capacity in brown adipose tissue of striped hamsters (*Cricetulus barabensis*) (Zhou et al. 2015), whereas field voles (*Microtus agrestis*) show little change in comparable oxidative measures (Selman et al. 2002). Cold exposure also reorganizes correlations among oxidative indicators across organs in *C. barabensis*, suggesting coordinated but tissue-dependent regulation (Chen et al. 2015). Brandt’s voles (*Lasiopodomys brandtii*) show similarly selective responses, with CAT increasing only in some tissues across thermal treatments (Xu et al. 2019). Seasonal studies in birds further show that increased winter thermoregulatory demand does not necessarily produce greater systemic oxidative damage (Jiménez et al. 2025). Together, these studies indicate that the relationship between metabolic demand, antioxidant defense, and oxidative damage is strongly tissue- and species-dependent.

The Yunnan red-backed vole (*Eothenomys miletus*) and Chevrier’s field mouse (*Apodemus chevrieri*) occur sympatrically in subalpine and montane shrub-meadow habitats of the Hengduan Mountains Region of China, where they experience pronounced diel and seasonal temperature variation (Zhu et al. 2008). The two species belong to different rodent families (Cricetidae and Muridae) and also differ in feeding ecology. *Eothenomys miletus* is a primarily herbivorous vole that feeds on roots, stems, and leaves of herbaceous plants, whereas *A. chevrieri* is an omnivorous field mouse with a broader diet that includes seeds, fruits, and arthropods (Zhang et al. 2020; Gong et al. 2021). These characteristics make the two species suitable for examining whether sympatric plateau rodents share a common physiological framework of cold acclimation while differing in the magnitude and tissue specificity of their responses.

Previous work has established substantial plasticity in body mass regulation and thermogenesis in both species. In *E. miletus*, cold acclimation and environmental variation alter energy metabolism, leptin signaling, and body-mass regulation (Zhu et al. 2010a, 2010b; Chen et al. 2022), and high-altitude or food-limitation responses are also associated with changes in gut microorganisms and physiological markers (Zhang et al. 2024). In *A. chevrieri*, cold, photoperiod, food availability, and season influence thermogenesis, energy intake, body mass, and digestive morphology (Zhu et al. 2011, 2012; Jia et al. 2023, Gao and Zhu 2024). Metabolomic variation across populations of *E. miletus* has additionally been documented in the Hengduan Mountains (Zhang et al. 2019). Despite this background, it remains unclear whether sustained cold-induced metabolic remodeling in these two species is accompanied by coordinated changes in antioxidant capacity and oxidative damage across multiple organs, and whether the structure of these oxidative responses differs between species.

We therefore exposed *E. miletus* and *A. chevrieri* to either 25°C ± 1°C (control) and 5°C ± 1°C (cold exposure) for 28 days under laboratory conditions. We measured body surface temperature, body mass, food intake, and RMR; quantified major-organ mass and digestive-tract morphology; and assayed SOD, CAT, MDA, and H_2_O_2_ in multiple tissues. We then used correlation analysis and principal component analysis (PCA) to characterize multiorgan patterns of oxidative regulation. Specifically, we asked whether long-term cold exposure (1) alters energy balance and thermoregulatory traits, (2) produces tissue-specific antioxidant and oxidative-damage responses, and (3) is associated with different patterns of inter-organ oxidative regulation in the two species.

## Materials and methods

### Animals and experimental design

Experimental protocols were approved by the Institutional Animal Care and Use Committee of the School of Life Sciences, Yunnan Normal University (No. 13-0901-011). *Eothenomys miletus* and *A. chevrieri* were collected in April 2025 from Kunming, Yunnan Province, China (24°52ʹ4″ N, 102°51ʹ57″ E, at 2020 m altitude). Field-captured animals were brought to the laboratory and acclimated for one month before the experiment. Throughout acclimation and the experiment, animals were housed individually in standard cages without bedding so that body mass and food intake could be measured for each individual. The colony room was maintained at 25°C ± 1°C under a 12 h light:12 h dark photoperiod (lights on at 08:00 and off at 20:00) with standard fluorescent illumination and no supplemental UV exposure. Food and water were provided *ad libitum*. Animals received a commercial standard rodent maintenance diet produced by Kunming Medical University. According to the manufacturer, the diet contained approximately 22% crude protein, 4% crude fat, 5% crude fiber, 8% crude ash, 1.0–1.8% calcium, and 0.6–1.2% phosphorus, with a metabolizable energy of approximately 17.5 kJ/g. These housing conditions approximated summer temperature at the collection locality and standardized thermal history before cold treatment.

Fourteen *E. miletus* and 14 *A. chevrieri* of similar body mass were randomly assigned to a control treatment (25°C ± 1°C, *n* = 7 per species; *E. miletus*: 4 males and 3 females, *A. chevrieri*: 5 males and 2 females) and a cold treatment (5°C ± 1°C, *n* = 7 per species; *E. miletus*: 5 males and 2 females, *A. chevrieri*: 6 males and 1 female). The exposure lasted 28 days. Endpoint-specific sample sizes differed because complete measurements were available for six individuals (Control treatment: *E. miletus*: 4 males and 2 females, *A. chevrieri*: 5 males and 1 female; cold treatment: *E. miletus*: 4 males and 2 females, *A. chevrieri*: 5 males and 1 female) per treatment for body mass, food intake, RMR, organ mass, and digestive-tract morphology, whereas body-temperature and biochemical measurements included seven individuals per treatment. Body mass, food intake, and RMR were assessed at baseline (0 day) and again near the end of exposure (28 days); the final measurements were completed 1–2 days before terminal tissue collection.

### Physiological measurements

#### Body surface temperature

Animal surface temperature was measured using an animal phenotype imaging system (WIC640-SUW; Workswell, Roznov, Czech Republic) at an imaging distance of 1 m, following the general thermal-imaging approach of Tattersall and Cadena (2010). The maximum body surface temperature obtained from each thermogram was used for statistical analysis.

#### Body mass and food intake

Body mass was measured with an LT502 electronic balance (accurate to 0.01 g). Animals were fed a measured quantity of food between 09:00 and 10:00. Remaining food, including residues in the feeding cage, was collected at the same time the following day. Daily food intake was calculated as the mass of food provided minus the mass remaining.

#### RMR

RMR was measured using an eight-channel FMS portable respirometry system (Sable Instruments, Las Vegas, NV, USA), following the general measurement procedure described by Hou et al. (2023). Oxygen consumption and carbon dioxide production (*V* O_2_/*V* CO_2_) were recorded as indices of metabolic rate. Before measurement, animals were allowed to the respirometry chamber for 30–60 min. After acclimation, data from a stable 15-min recording period were used for subsequent analysis.

#### Organ mass and digestive-tract morphology

On day 28, animals were euthanized with isoflurane. The brain, heart, liver, spleen, lungs, kidneys, and hind-limb skeletal muscles were rapidly dissected. The stomach, large intestine, small intestine, and cecum were then isolated. Attached connective tissues and luminal contents were removed, and tissues were blotted dry with filter paper. Organ mass and digestive-tract length were measured before tissues were rapidly frozen in liquid nitrogen for storage.

#### Determination of antioxidant capacity in tissues

Tissues were homogenized in pre-cooled 0.9% NaCl at a tissue-to-solution ratio of 1:9. Homogenates were centrifuged at 3000 r/min for 15 min, and the supernatants were used for biochemical assays. SOD activity was measured using the xanthine oxidase (hydroxylamine) method with 0.1 mL of 10% homogenate and the absorbance at 550 nm; activity was expressed as nmol/g protein. CAT activity was measured in a 2.2 mL reaction system containing 0.1 mL of 1% homogenate, with absorbance measured at 405 nm. One unit of CAT activity was defined as the amount decomposing 1 μmol H_2_O_2_ per mg tissue protein per second, and activity was expressed as μmol/g protein. MDA was measured in a 4.2 mL reaction system containing 0.1 mL of 10% homogenate at 532 nm and expressed as nmol/g protein. H_2_O_2_ was measured in a 2.1 mL reaction system containing 0.1 mL of 10% homogenate at 405 nm and expressed as μmol/g protein. Commercial assay kits were obtained from Nanjing Jiancheng Bioengineering Institute and used according to the manufacturer’s instructions. Protein concentration in each supernatant was determined by the Folin-phenol method using bovine serum albumin as the standard.

### Statistical and data processing

Statistical analyses of physiological, morphological, and biochemical endpoints were performed in GraphPad Prism 10.1. For body surface temperature and all day-28 organ, digestive-tract, and biochemical endpoints, ordinary two-way analysis of variance (ANOVA) included species (*E. miletus vs. A. chevrieri*), temperature treatment (25°C vs. 5°C), and the species × temperature interaction. Sex was not included as an experimental factor because the study was not designed or powered for sex-stratified inference. Body mass, food intake, and RMR at 0 day and 28 days were analyzed separately; day was not included as a factor in a single repeated-measures model. At each day, these variables were analyzed using the same species × temperature two-way ANOVA. The omnibus species × temperature interaction is reported in the Results as the ANOVA *F* statistic with numerator and residual degrees of freedom and the exact *P* value. When appropriate, Šídák-adjusted simple-effects comparisons were used to compare cold and control treatments within each species. The one-degree-of-freedom simple effects are expressed as *F*_(1, residual df)_ = *t*², using the corresponding *t* statistic, together with the exact Šídák-adjusted *P* value. This *F*-equivalent reporting of simple contrasts is distinct from, and does not replace, the omnibus species × temperature interaction test. Normality and homogeneity of variance were evaluated using the Shapiro–Wilk and Levene tests, respectively. Pearson correlations were calculated in OriginPro 2024. For each correlation matrix, the complete set of pairwise raw *P* values was adjusted using the Benjamini–Hochberg false discovery rate (FDR) procedure, and statistical significance was determined from the FDR-adjusted *P* values. PCA was performed in OriginPro 2024 using standardized *z*-scores of SOD, CAT, MDA, and H_2_O_2_. In the first PCA, organs (brain, heart, liver, lungs, kidneys, small intestine, and skeletal muscle) for each species and treatment were treated as observations. A complementary PCA examined the loading structure of the oxidative indicators. Data are presented as mean ± SEM, and FDR-adjusted *P* < 0.05 was considered significant for correlation analyses.

## Results

### Effects of cold exposure on body surface temperature, body mass, food intake, and RMR

Cold exposure altered thermoregulatory and metabolic traits in both species. For maximum body surface temperature, the species × temperature interaction was not significant (*F*_(1, 24)_ = 0.133, *P* = 0.719). Šídák-adjusted simple effects comparisons showed lower surface temperature in the cold treatment for both *E. miletus* (*F*_(1, 24)_ = 22.712, *P* = 0.000) and *A. chevrieri* (*F*_(1, 24)_ = 18.066, *P* = 0.001) (Fig. 1A, B). At 0 day, the species × temperature interactions were not significant for body mass (*F*_(1, 20)_ = 0.000, *P* = 0.989), food intake (*F*_(1, 20)_ = 0.009, *P* = 0.924), or RMR (*F*_(1, 20)_ = 0.009, *P* = 0.926). Cold and control groups also did not differ within either species at baseline: body mass, *E. miletus* (*F*_(1, 20)_ = 0.013, *P* = 0.992) and *A. chevrieri* (*F*_(1, 20)_ = 0.009, *P* = 0.994); food intake, *E. miletus* (*F*_(1, 20)_ = 0.162, *P* = 0.905) and *A. chevrieri* (*F*_(1, 20)_ = 0.071, *P* = 0.957); and RMR, *E. miletus* (*F*_(1, 20)_ = 0.147, *P* = 0.913) and *A. chevrieri* (*F*_(1, 20)_ = 0.063, *P* = 0.962) (Fig. 1C). At 28 days, species × temperature interactions remained nonsignificant for body mass (*F*_(1, 20)_ = 0.441, *P* = 0.514), food intake (*F*_(1, 20)_ = 3.695, *P* = 0.069), and RMR (*F*_(1, 20)_ = 0.562, *P* = 0.462). Within each species, cold- exposed animals had lower body mass [*E. miletus* (*F*_(1, 20)_ = 18.911, *P* = 0.001); *A. chevrieri* (*F*_(1, 20)_ = 11.623, *P* = 0.006)] but higher food intake [*E. miletus* (*F*_(1, 20)_ = 139.215, *P* = 0.000); *A. chevrieri* (*F*_(1, 20)_ = 82.455, *P* = 0.000)] and RMR [*E. miletus* (*F*_(1, 20)_ = 39.561, *P* = 0.000); *A. chevrieri* (*F*_(1, 20)_ = 27.350, *P* = 0.000)] than controls (Fig. 1C).

**Fig. 1.**
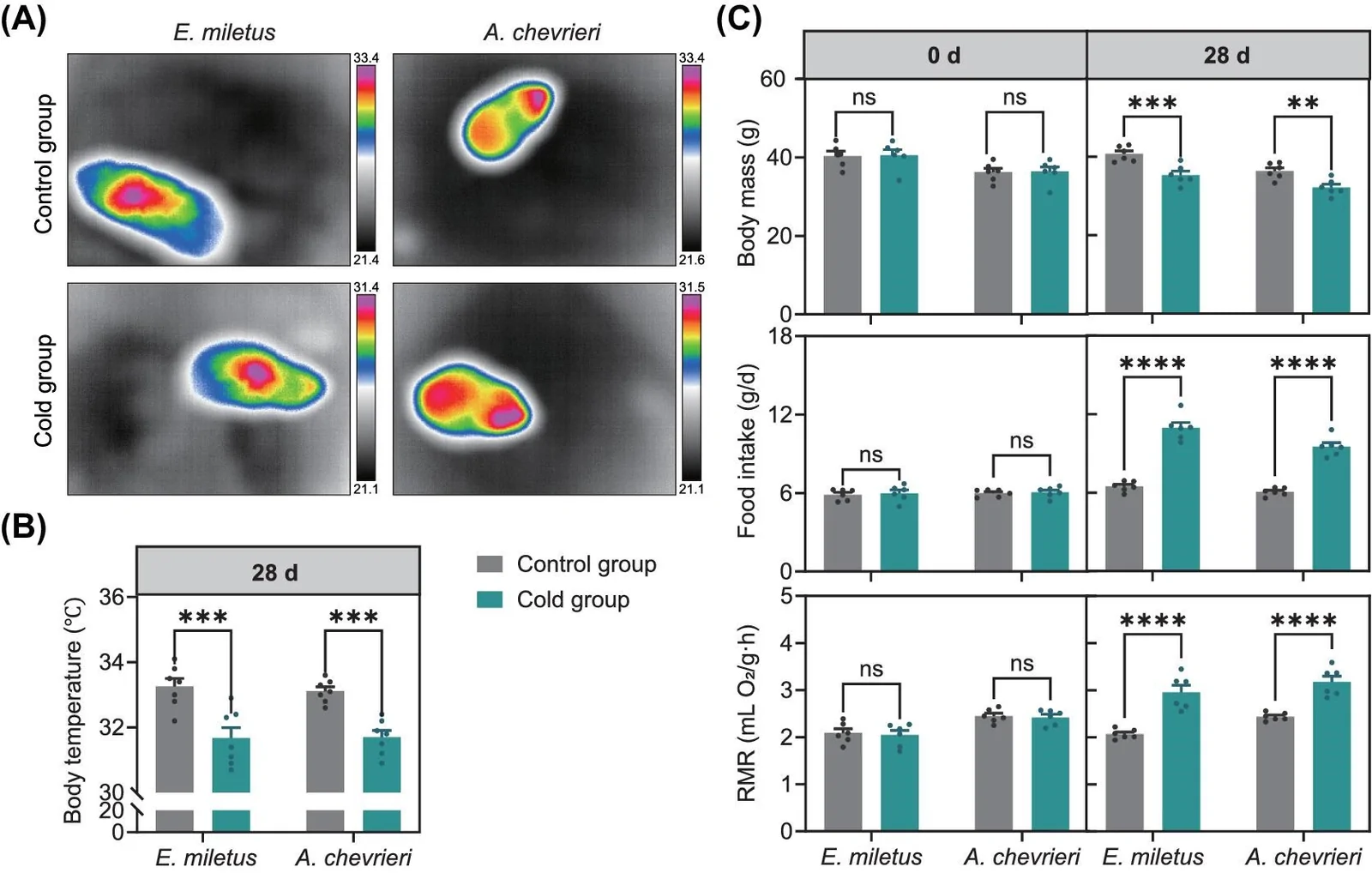
Effects of cold exposure on body surface temperature, body mass, food intake, and RMR in *E. miletus* and *A. chevrieri*. (A) Representative infrared thermograms for control and cold-treated animals. (B) Maximum body surface temperature at 28 days. (C) Body mass, food intake, and RMR at 0 day and 28 days. Data are expressed as mean ± s.e.m., ns indicates no significant difference, two stars (**), three stars (***), and four stars (****) indicate *P* < 0.01, *P* < 0.001, *P* < 0.0001, respectively.

### Effects of cold exposure on the morphology of major organs and the digestive tract

Among the major organs examined, liver mass was the only trait that showed a consistent cold-exposure effect in both species. The species × temperature interaction was not significant for brain (*F*_(1, 20)_ = 0.003, *P* = 0.955), heart (*F*_(1, 20)_ = 0.000, *P* = 1.000), liver (*F*_(1, 20)_ = 0.625, *P* = 0.438), spleen (*F*_(1, 20)_ = 0.000, *P* = 1.000), lungs (*F*_(1, 20)_ = 0.000, *P* = 1.000), or kidneys (*F*_(1, 20)_ = 0.037, *P* = 0.850). Šídák-adjusted simple effects showed that liver mass increased in both *E. miletus* (*F*_(1, 20)_ = 39.928, *P* = 0.000) and *A. chevrieri* (*F*_(1, 20)_ = 27.045, *P* = 0.000) (Fig. 2A). In contrast, brain mass did not differ between treatments in *E. miletus* (*F*_(1, 20)_ = 0.233, *P* = 0.866) or *A. chevrieri* (*F*_(1, 20)_ = 0.317, *P* = 0.823); nor did heart mass [*E. miletus* (*F*_(1, 20)_ = 0.098, *P* = 0.941); *A. chevrieri* (*F*_(1, 20)_ = 0.098, *P* = 0.941)], spleen mass [*E. miletus* (*F*_(1, 20)_ = 4.549, *P* = 0.089); *A. chevrieri* (*F*_(1, 20)_ = 4.549, *P* = 0.089)], lung mass [*E. miletus* (*F*_(1, 20)_ = 0.128, *P* = 0.924); *A. chevrieri* (*F*_(1, 20)_ = 0.128, *P* = 0.924)], or kidney mass [*E. miletus* (*F*_(1, 20)_ = 0.819, *P* = 0.611); *A. chevrieri* (*F*_(1, 20)_ = 0.401, *P* = 0.783)] (Fig. 2A).

**Fig. 2.**
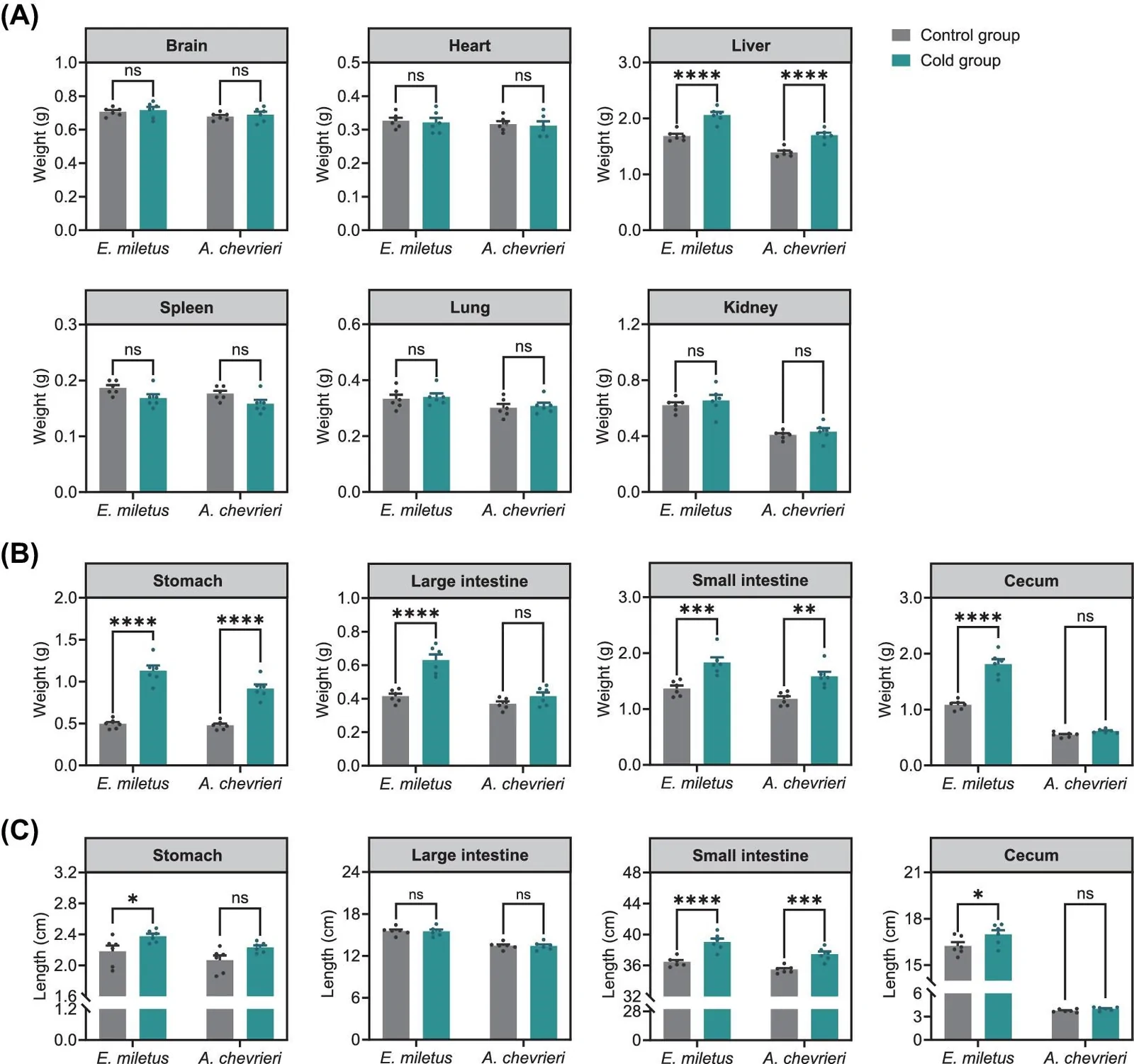
Effects of cold exposure on major organs and the digestive tract in *E. miletus* and *A. chevrieri*. (A) Mass of the brain, heart, liver, spleen, lungs, and kidneys. (B) Mass of the stomach, large intestine, small intestine, and cecum. (C) Length of the stomach, large intestine, small intestine, and cecum. Data are represented as mean ± s.e.m., ns indicates no significant difference, one star (*), two stars (**), three stars (***), and four stars (****) indicate *P* < 0.05, *P* < 0.01, *P* < 0.001, *P* < 0.0001, respectively.

Cold exposure also induced morphological changes in the digestive tract, with more extensive changes in the *E. miletus* than in *A. chevrieri*. For digestive-tract mass, species × temperature interactions were significant for the stomach (*F*_(1, 20)_ = 5.161, *P* = 0.034), large intestine (*F*_(1, 20)_ = 13.611, *P* = 0.002), and cecum (*F*_(1, 20)_ = 47.740, *P* = 0.000), but not the small intestine (*F*_(1, 20)_ = 0.187, *P* = 0.670). In *E. miletus*, the mass of the stomach (*F*_(1, 20)_ = 108.874, *P* = 0.000), large intestine (*F*_(1, 20)_ = 43.540, *P* = 0.000), small intestine (*F*_(1, 20)_ = 21.365, *P* = 0.000), and cecum (*F*_(1, 20)_ = 115.690, *P* = 0.000) increased under cold conditions (Fig. 2B). In *A. chevrieri*, stomach (*F*_(1, 20)_ = 52.152, *P* = 0.000) and small intestine mass (*F*_(1, 20)_ = 16.092, *P* = 0.001) increased, whereas large intestine (*F*_(1, 20)_ = 1.907, *P* = 0.332) and cecum mass (*F*_(1, 20)_ = 0.969, *P* = 0.560) did not change significantly. For digestive-tract length, none of the species × temperature interactions was significant: stomach (*F*_(1, 20)_ = 0.088, *P* = 0.770), large intestine (*F*_(1, 20)_ = 0.000, *P* = 0.992), small intestine (*F*_(1, 20)_ = 0.836, *P* = 0.371), and cecum (*F*_(1, 20)_ = 1.663, *P* = 0.212). In *E. miletus*, the stomach (*F*_(1, 20)_ = 6.791, *P* = 0.034), small intestine (*F*_(1, 20)_ = 31.330, *P* = 0.000), and cecum (*F*_(1, 20)_ = 7.485, *P* = 0.025) were longer after cold exposure, whereas large intestine length was unchanged (*F*_(1, 20)_ = 0.017, *P* = 0.990) (Fig. 2C). In *A. chevrieri*, only the small intestine increased significantly in length (*F*_(1, 20)_ = 18.526, *P* = 0.001); stomach (*F*_(1, 20)_ = 4.780, *P* = 0.080), large intestine (*F*_(1, 20)_ = 0.013, *P* = 0.992), and cecum length (*F*_(1, 20)_ = 0.832, *P* = 0.606) were unchanged.

### Effects of cold exposure on the antioxidant system and oxidative stress markers

Cold exposure produced tissue- and species-specific changes in SOD activity (Fig. 3A). Species × temperature interactions were significant in all seven tissues: brain (*F*_(1, 24)_ = 46.272, *P* = 0.000); heart (*F*_(1, 24)_ = 63.625, *P* = 0.000); liver (*F*_(1, 24)_ = 155.687, *P* = 0.000); lungs (*F*_(1, 24)_ = 50.628, *P* = 0.000); kidneys (*F*_(1, 24)_ = 12.870, *P* = 0.002); small intestine (*F*_(1, 24)_ = 6.708, *P* = 0.016); skeletal muscle (*F*_(1, 24)_ = 7.281, *P* = 0.013). In *E. miletus*, SOD activity increased in the brain (*F*_(1, 24)_ = 73.244, *P* = 0.000), heart (*F*_(1, 24)_ = 86.410, *P* = 0.000), liver (*F*_(1, 24)_ = 183.409, *P* = 0.000), kidneys (*F*_(1, 24)_ = 11.987, *P* = 0.004), small intestine (*F*_(1, 24)_ = 34.499, *P* = 0.000), and skeletal muscle (*F*_(1, 24)_ = 17.227, *P* = 0.001), but decreased in the lungs (*F*_(1, 24)_ = 125.680, *P* = 0.000). In *A. chevrieri*, SOD activity decreased in the liver (*F*_(1, 24)_ = 16.834, *P* = 0.001) and increased in the small intestine (*F*_(1, 24)_ = 90.942, *P* = 0.000). No significant cold-versus-control difference was detected in the brain (*F*_(1, 24)_ = 1.127, *P* = 0.509), heart (*F*_(1, 24)_ = 3.940, *P* = 0.114), lungs (*F*_(1, 24)_ = 1.318, *P* = 0.456), kidneys (*F*_(1, 24)_ = 2.596, *P* = 0.226), or skeletal muscle (*F*_(1, 24)_ = 0.112, *P* = 0.933) of *A. chevrieri*.

**Fig. 3.**
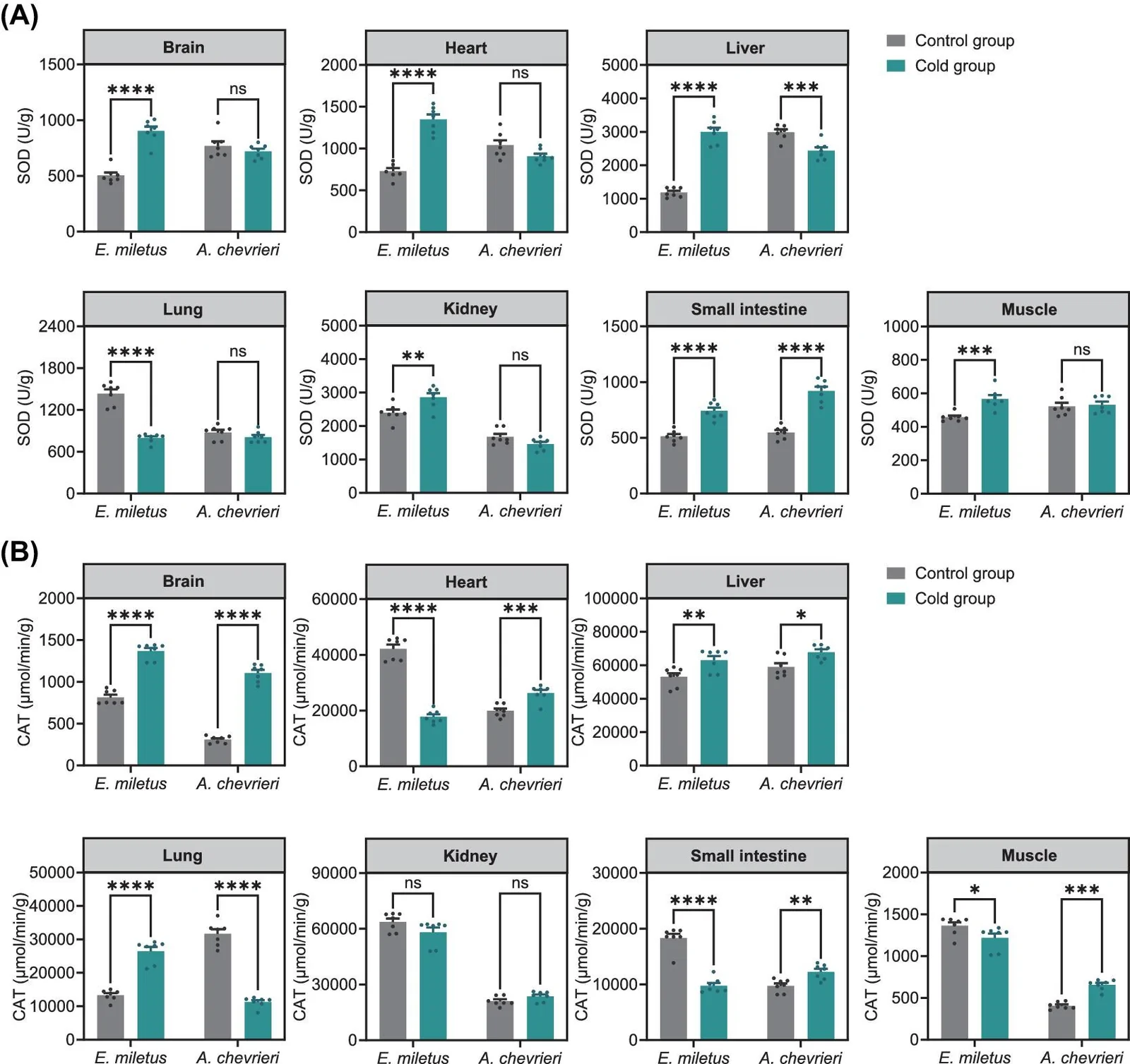
Effects of cold exposure on SOD and CAT activity in major organs and tissues of *E. miletus* and *A. chevrieri*. (A) SOD activity in the brain, heart, liver, lungs, kidneys, small intestine, and muscle tissue. (B) CAT activity in the same tissues. Data are expressed as mean ± s.e.m., ns indicates no significant difference, one star (*), two stars (**), three stars (***), and four stars (****) indicate *P* < 0.05, *P* < 0.01, *P* < 0.001, *P* < 0.0001, respectively.

CAT activity also exhibited marked tissue-specific responses to cold exposure (Fig. 3B). Species × temperature interactions were significant in the brain (*F*_(1, 24)_ = 14.134, *P* = 0.001), heart (*F*_(1, 24)_ = 192.921, *P* = 0.000), lungs (*F*_(1, 24)_ = 273.483, *P* = 0.000), kidneys (*F*_(1, 24)_ = 5.487, *P* = 0.028), small intestine (*F*_(1, 24)_ = 94.143, *P* = 0.000), and skeletal muscle (*F*_(1, 24)_ = 29.624, *P* = 0.000), but not in the liver (*F*_(1, 24)_ = 0.062, *P* = 0.806). In *E. miletus*, CAT activity increased in the brain (*F*_(1, 24)_ = 150.283, *P* = 0.000), liver (*F*_(1, 24)_ = 10.565, *P* = 0.007), lungs (*F*_(1, 24)_ = 83.984, *P* = 0.000), and small intestine (*F*_(1, 24)_ = 112.161, *P* = 0.000), but decreased in the heart (*F*_(1, 24)_ = 241.912, *P* = 0.000) and skeletal muscle (*F*_(1, 24)_ = 7.966, *P* = 0.019); kidney CAT activity was unchanged (*F*_(1, 24)_ = 5.124, *P* = 0.065). In *A. chevrieri*, CAT activity increased in the brain (*F*_(1, 24)_ = 308.906, *P* = 0.000), heart (*F*_(1, 24)_ = 16.722, *P* = 0.001), liver (*F*_(1, 24)_ = 8.400, *P* = 0.016), small intestine (*F*_(1, 24)_ = 9.804, *P* = 0.009), and skeletal muscle (*F*_(1, 24)_ = 23.764, *P* = 0.000), decreased in the lungs (*F*_(1, 24)_ = 202.294, *P* = 0.000), and did not change significantly in the kidneys (*F*_(1, 24)_ = 1.101, *P* = 0.516).

Cold exposure produced tissue- and species-specific changes in MDA (Fig. 4A). Species × temperature interactions were significant in the heart (*F*_(1, 24)_ = 32.476, *P* = 0.000), liver (*F*_(1, 24)_ = 97.771, *P* = 0.000), lungs (*F*_(1, 24)_ = 40.208, *P* = 0.000), kidneys (*F*_(1, 24)_ = 9.577, *P* = 0.005), small intestine (*F*_(1, 24)_ = 13.777, *P* = 0.001), and skeletal muscle (*F*_(1, 24)_ = 50.886, *P* = 0.000), but not in the brain (*F*_(1, 24)_ = 1.187, *P* = 0.287). In *E. miletus*, MDA decreased in the brain (*F*_(1, 24)_ = 12.775, *P* = 0.003) and lungs (*F*_(1, 24)_ = 142.514, *P* = 0.000), increased in the heart (*F*_(1, 24)_ = 34.082, *P* = 0.000), small intestine (*F*_(1, 24)_ = 103.321, *P* = 0.000), and skeletal muscle (*F*_(1, 24)_ = 104.380, *P* = 0.000), and did not change significantly in the liver (*F*_(1, 24)_ = 1.588, *P* = 0.391) or kidneys (*F*_(1, 24)_ = 0.021, *P* = 0.987). In *A. chevrieri*, MDA increased in the liver (*F*_(1, 24)_ = 232.372, *P* = 0.000) and small intestine (*F*_(1, 24)_ = 24.163, *P* = 0.000), decreased in the lungs (*F*_(1, 24)_ = 8.824, *P* = 0.013) and kidneys (*F*_(1, 24)_ = 17.896, *P* = 0.001), and did not change significantly in the brain (*F*_(1, 24)_ = 4.134, *P* = 0.104), heart (*F*_(1, 24)_ = 4.934, *P* = 0.071), or skeletal muscle (*F*_(1, 24)_ = 0.016, *P* = 0.990).

**Fig. 4.**
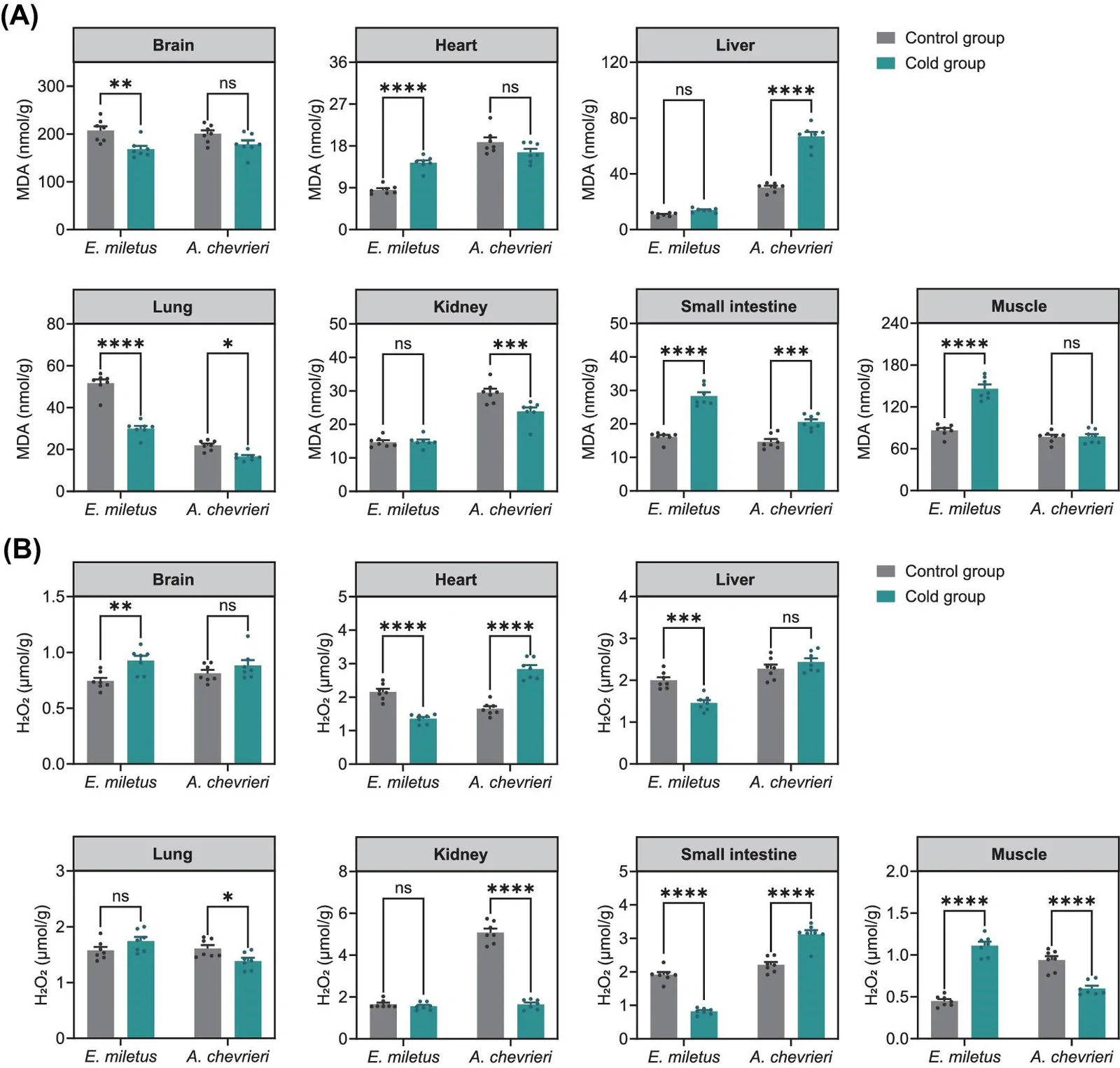
Effects of cold exposure on MDA and H_2_O_2_ levels in major organs and tissues of *E. miletus* and *A. chevrieri*. (A) MDA content. (B) H_2_O_2_ content. Data are expressed as mean ± s.e.m., ns indicates no significant difference, one star (*), two stars (**), three stars (***), and four stars (****) indicate *P* < 0.05, *P* < 0.01, *P* < 0.001, *P* < 0.0001, respectively.

H_2_O_2_ levels likewise exhibited tissue-specific responses to cold exposure (Fig. 4B). Species × temperature interactions were significant in the heart (*F*_(1, 24)_ = 131.002, *P* = 0.000), liver (*F*_(1, 24)_ = 17.224, *P* = 0.000), lungs (*F*_(1, 24)_ = 9.367, *P* = 0.005), kidneys (*F*_(1, 24)_ = 202.617, *P* = 0.000), small intestine (*F*_(1, 24)_ = 140.065, *P* = 0.000), and skeletal muscle (*F*_(1, 24)_ = 170.562, *P* = 0.000), but not in the brain (*F*_(1, 24)_ = 2.259, *P* = 0.146). In *E. miletus*, H_2_O_2_ increased in the brain (*F*_(1, 24)_ = 11.620, *P* = 0.005) and skeletal muscle (*F*_(1, 24)_ = 148.898, *P* = 0.000), decreased in the heart (*F*_(1, 24)_ = 42.473, *P* = 0.000), liver (*F*_(1, 24)_ = 20.468, *P* = 0.000), and small intestine (*F*_(1, 24)_ = 83.068, *P* = 0.000), and did not change significantly in the lungs (*F*_(1, 24)_ = 3.398, *P* = 0.149) or kidneys (*F*_(1, 24)_ = 0.388, *P* = 0.788). In *A. chevrieri*, H_2_O_2_ increased in the heart (*F*_(1, 24)_ = 93.497, *P* = 0.000) and small intestine (*F*_(1, 24)_ = 58.109, *P* = 0.000), decreased in the lungs (*F*_(1, 24)_ = 6.175, *P* = 0.040), kidneys (*F*_(1, 24)_ = 430.695, *P* = 0.000), and skeletal muscle (*F*_(1, 24)_ = 39.278, *P* = 0.000), and did not change significantly in the brain (*F*_(1, 24)_ = 1.646, *P* = 0.379) or liver (*F*_(1, 24)_ = 1.809, *P* = 0.346).

### Inter-organ correlations among oxidative stress indicators

To assess coordination of oxidative responses across organs, we calculated Pearson correlations separately for each species and oxidative indicator and applied Benjamini–Hochberg FDR correction within each correlation matrix. In *E. miletus*, CAT activity showed significant positive correlations among several organ between the heart and small intestine (FDR-adjusted *P* = 0.035), between the liver and kidneys (*P* = 0.040) and skeletal muscle (*P* = 0.040), between the lungs and kidneys (*P* = 0.001) and skeletal muscle (*P* = 0.005), and between the kidneys and skeletal muscle (*P* = 0.000) (Fig. 5C). No significant inter-organ correlations were detected for SOD, MDA, or H_2_O_2_ after FDR correction (Fig. 5A, E, G). In *A. chevrieri*, MDA showed a significant negative correlation between the brain and liver (FDR-adjusted *P* = 0.047; Fig. 5F), whereas SOD, CAT, and H_2_O_2_ showed no significant inter-organ correlations (Fig. 5B, D, H).

**Fig. 5.**
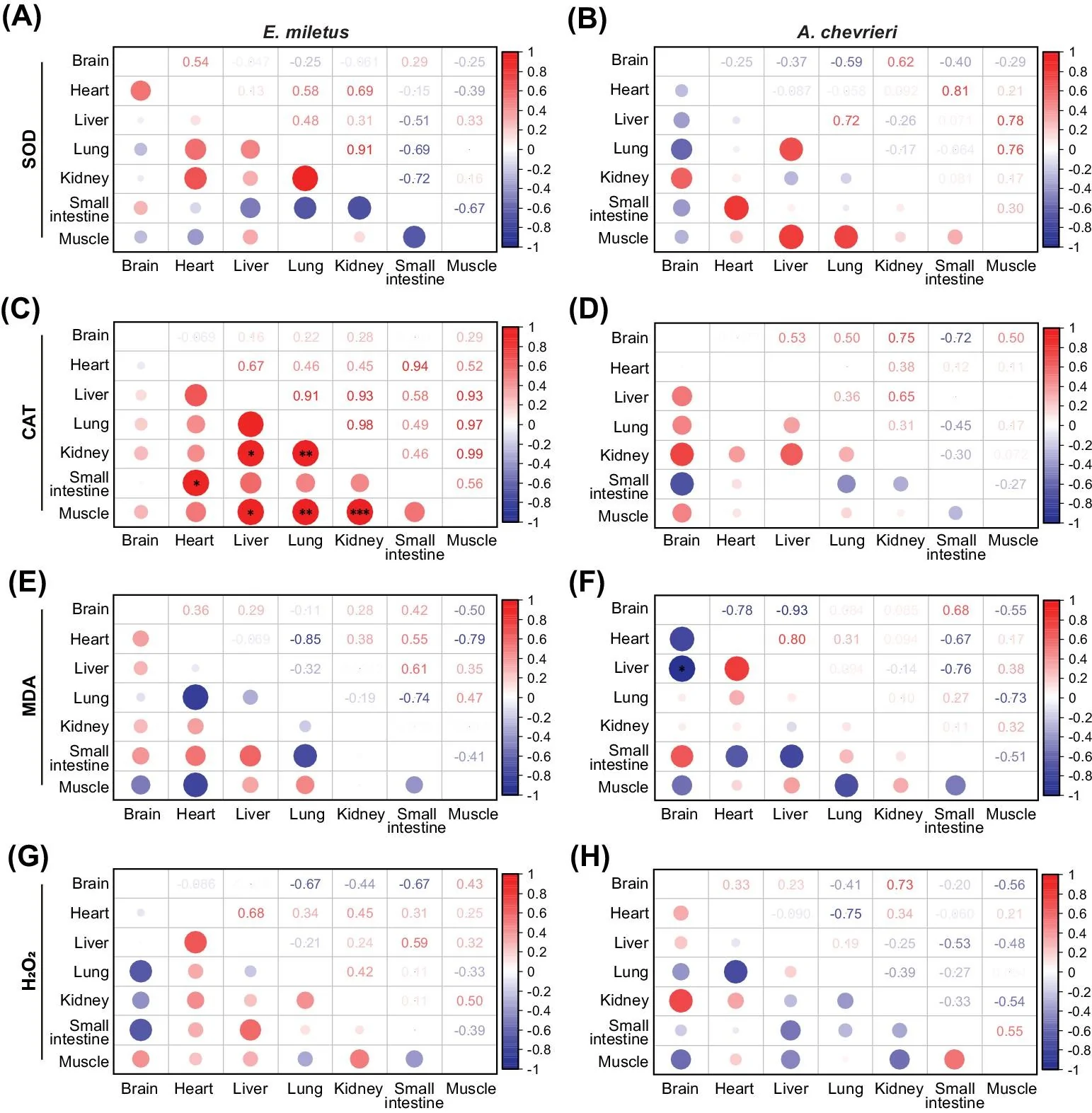
Inter-organ correlations of SOD, CAT, MDA, and H_2_O_2_ in *E. miletus* and *A. chevrieri*. (A, C, E, G) Correlation matrices for SOD, CAT, MDA, and H_2_O_2_, respectively, in *E. miletus*. (B, D, F, H) Corresponding matrices for *A. chevrieri*. Significance is based on Benjamini–Hochberg FDR‑adjusted *P* values within each matrix. One star (*), two stars (**), and three stars (***) indicate *P* < 0.05, *P* < 0.01, *P* < 0.001, respectively.

Within individual organs, correlations among SOD, CAT, MDA, and H_2_O_2_ were generally weak after FDR correction. The only significant association was a positive correlation between SOD and CAT activity in the brain of *A. chevrieri* (FDR-adjusted *P* = 0.032; Fig. 6B). No other within-organ correlations were significant in either species (Fig. 6).

**Fig. 6.**
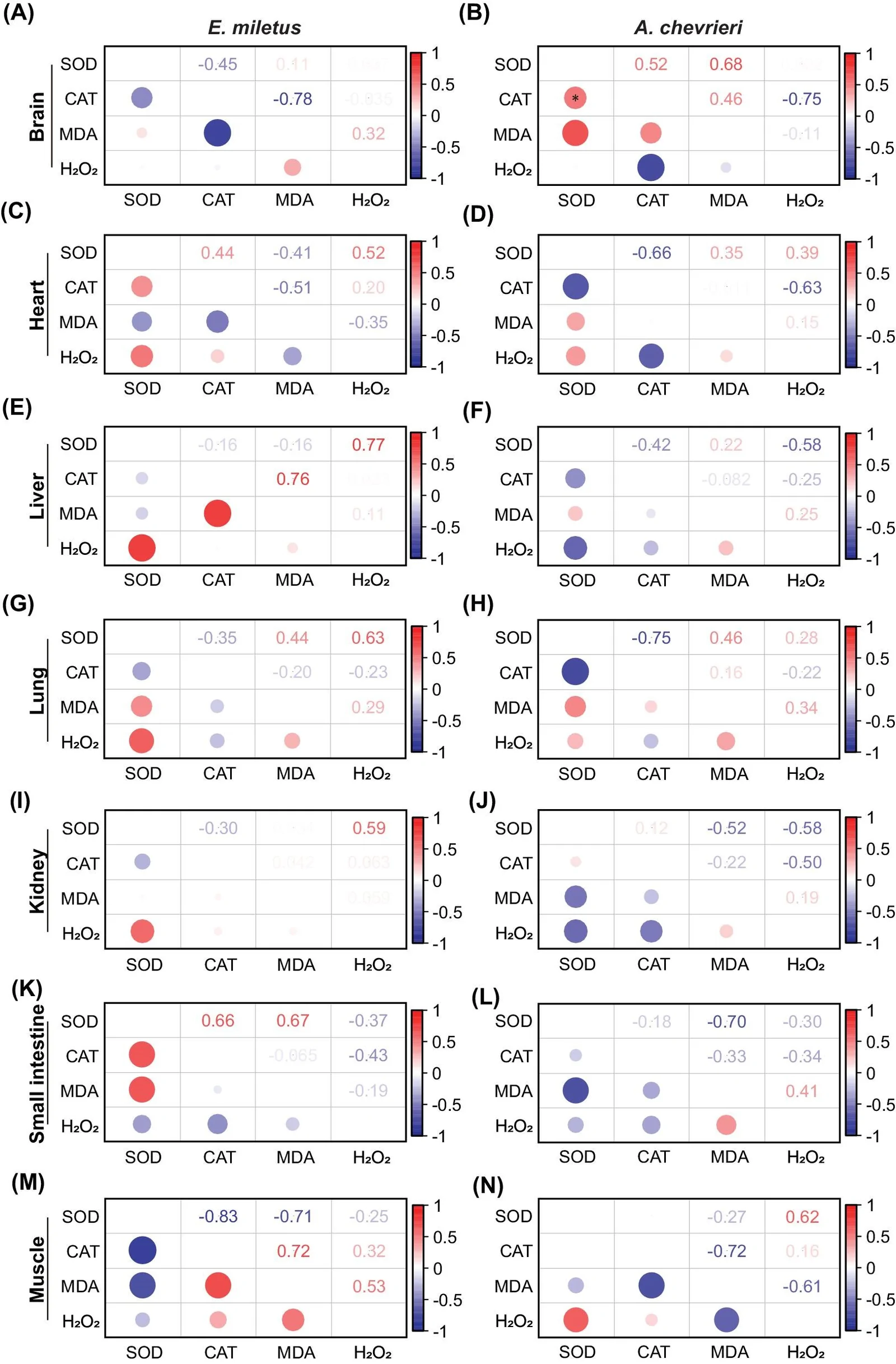
Within-organ correlations among SOD, CAT, MDA, and H_2_O_2_ in *E. miletus* and *A. chevrieri*. (A, C, E, G, I, K, M) Correlations within the brain, heart, liver, lungs, kidneys, small intestine, and skeletal muscle, respectively, of *E. miletus*. (B, D, F, H, J, L, N) Corresponding correlations in *A. chevrieri*. Significance is based on Benjamini–Hochberg FDR‑adjusted *P* values within each matrix. One star (*) indicates *P* < 0.05.

### PCA of oxidative stress indicators in major organs

Two complementary PCAs were used to summarize multivariate patterns in SOD, CAT, MDA, and H_2_O_2_. In the organ-based PCA, liver and small intestine scores were separated from those of the brain and skeletal muscle primarily along PC1 (Fig. 7A), indicating that digestive and metabolic organs occupied distinct regions of the multivariate oxidative-stress space. Scores for *E. miletus* and *A. chevrieri* overlapped substantially for several organs, including the brain, heart, liver, lungs, and skeletal muscle, whereas the kidney and small intestine showed greater dispersion between species and treatments. The directions associated with antioxidant enzymes (SOD and CAT) differed from those associated with oxidative-damage markers (MDA and H_2_O_2_), consistent with partially distinct antioxidant and damage axes.

**Fig. 7.**
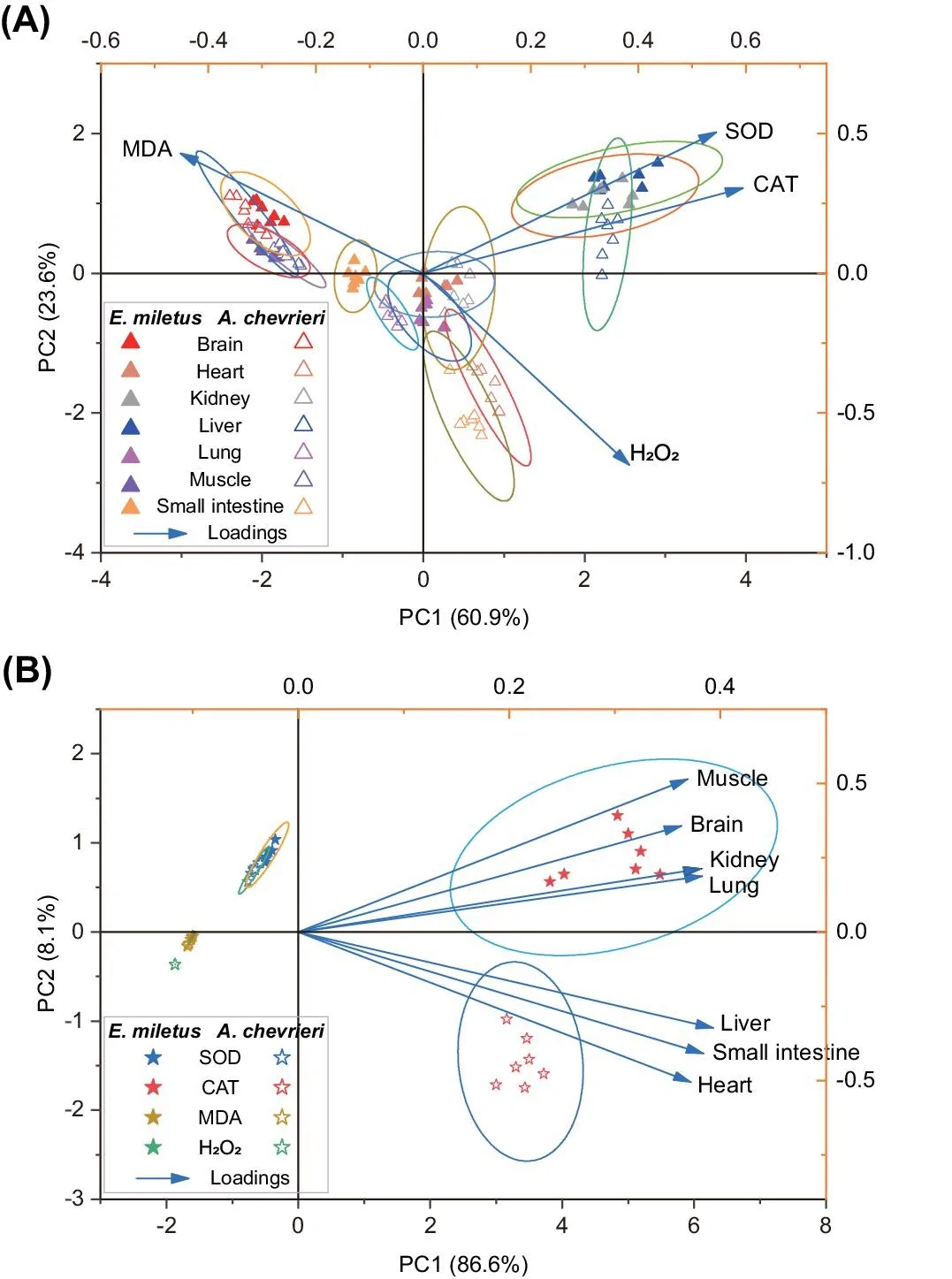
PCA analysis of oxidative-stress indicators in *E. miletus* and *A. chevrieri*. (A) PCA of organ observations in the multivariate space defined by standardized SOD, CAT, MDA, and H_2_O_2_ values. Each point represents the centroid of an organ for a given species and temperature treatment; ellipses show 95% confidence regions. (B) Complementary PCA summarizing loading patterns of the organs across oxidative indicators and species. Solid and open symbols denote *E. miletus* and *A. chevrieri*, respectively.

In the complementary loading-based PCA, organ-related loadings were positive along PC1, whereas PC2 separated skeletal muscle, brain, kidneys, and lungs from liver, small intestine, and heart (Fig. 7B). CAT-related loadings also occupied different regions of PC2 for the two species, consistent with species-specific organization of CAT responses across organs. These PCA patterns are descriptive and were used to visualize multivariate structure rather than to establish causal relationships among organs.

## Discussion

After 28 days of cold exposure, both *E. miletus* and *A. chevrieri* had lower body mass and higher food intake and RMR than their respective controls. This pattern is consistent with the increase in energy intake and thermogenesis commonly observed during cold acclimation in small mammals (Wang and Wang 1996, Gordon 2012). Brandt’s voles similarly increase energy intake and thermogenic expenditure during cold exposure (Xu et al. 2019), and desert hamsters increase thermogenic capacity when acclimated to low temperature (Chi and Wang 2011). The reduction in body mass observed here differs from that reported for *C. barabensis* during high-fat diet refeeding after cold exposure, where increased energy expenditure limited fat accumulation without producing the same overall pattern of body mass loss (Huo et al. 2022). These comparisons emphasize that small mammals can meet the energetic demands of cold through different combinations of intake, expenditure, and body mass adjustment.

Cold exposure also increased liver mass in both species and produced marked digestive-tract remodeling, particularly in *E. miletus*. Increased digestive capacity can support the higher nutrient flux required for sustained thermogenesis. Comparable remodeling of digestive tissues and oxidative status occurs in *C. barabensis* during moderate cold acclimation (Chen et al. 2014), while the winter phenotype of Siberian hamsters (*Phodopus sungorus*) is accompanied by coordinated changes in energy balance and antioxidant defense (Jefimow et al. 2020). Other rodents show more limited structural responses, with temperature affecting brown adipose tissue and selected organs without extensive digestive-tract remodeling (Wang et al. 2015, Skrzep-Poloczek et al. 2017, Xu et al. 2019). The more extensive digestive remodeling observed in *E. miletus* suggests that gastrointestinal plasticity may make a greater contribution to supporting elevated energy turnover in this species.

Antioxidant responses were highly tissue specific and differed between species. *Eothenomys miletus* increased SOD and/or CAT activity in several organs, whereas *A. chevrieri* showed a broader pattern of CAT upregulation but more restricted SOD responses. Tissue-specific changes in SOD and CAT have also been reported during temperature acclimation in *C. barabensis* and seasonal acclimatization in *P. sungorus* (Chen et al. 2014, Jefimow et al. 2020). Long-term cold or cryotherapy models in rats likewise show that antioxidant enzymes can increase in some tissues while decreasing or remaining unchanged in others (Skrzep-Poloczek et al. 2017). Thus, cold exposure appears to reconfigure antioxidant capacity across organs rather than simply increasing antioxidant activity throughout the body.

Changes in MDA and H_2_O_2_ further showed that activation of antioxidant enzymes did not uniformly prevent the accumulation of oxidative stress or damage. Both rodents exhibited increases in oxidative-damage markers in some tissues and decreases in others. Similar organ-specific patterns have been reported in *L. brandtii*, in which thermal stress altered oxidative indices in selected organs while leaving others relatively stable (Xu et al. 2019). Chronic cold stress can also increase myocardial oxidative injury in rodents, particularly when antioxidant capacity is insufficient to match metabolic demand (Lv et al. 2023). In contrast, milder or intermittent cold exposure can increase antioxidant activity while reducing lipid peroxidation in some tissues (Wang et al. 2015, Skrzep-Poloczek et al. 2017). The present results therefore support a model in which sustained cold exposure produces a heterogeneous pattern of effective antioxidant compensation and residual oxidative stress across organs.

The correlation analyses provide a complementary view of this tissue-specific regulation. In *E. miletus*, CAT activity showed a comparatively dense pattern of significant positive inter-organ correlations among several metabolically active organs, whereas *A. chevrieri* showed only a few significant inter-organ or within-organ associations. Coordinated antioxidant regulation across organs has been described in animals undergoing major metabolic transitions, including hibernation and rewarming (Shemarova and Nikitina 2026), and oxidative state can also covary with energy metabolism, immune function, and thermoregulatory behavior (Sukhchuluun et al. 2018, Jiménez et al. 2025). Predictive and reactive antioxidant strategies likewise differ among heterothermic rodents (Jefimow et al. 2020). However, our experiment included one population per species and only one cold and one control temperature. The observed CAT-centered network in *E. miletus* and the comparatively sparse and localized correlation pattern observed in *A. chevrieri* should therefore be interpreted as different within-lifetime acclimation responses, not as evidence that phylogeny or dietary niche independently caused the difference.

## Conclusion

In conclusion, sustained cold exposure altered energy balance, digestive morphology, and oxidative regulation in both *E. miletus* and *A. chevrieri*. Both species exhibited a common response characterized by increased metabolic demand and tissue-specific remodeling of antioxidant defenses, but they differed in the organs involved and in the structure of inter-organ oxidative associations. *Eothenomys miletus* showed more extensive digestive remodeling and a denser CAT-centered correlation network, whereas *A. chevrieri* showed fewer significant inter-organ associations. Because the study compared one population of each species at only two laboratory temperatures, these differences should be interpreted as species-specific acclimation patterns rather than direct evidence of adaptive divergence attributable to phylogeny or feeding ecology. The experiment also used a single cold intensity, four oxidative indicators, and sex was not analyzed as an independent experimental factor. Future work across multiple temperatures, seasons, populations, and sexes, together with broader antioxidant and inflammatory pathways and gut-microbiome measurements, will be needed to determine how general these patterns are in alpine small mammals.

## Data Availability

The data associated with this article are available at https://doi.org/10.6084/m9.figshare.31803784.
